# Factors associated with mobile app‐based ordering of HIV self‐test kits among men who have sex with men in Atlanta, Detroit and New York City: an exploratory secondary analysis of a randomized control trial

**DOI:** 10.1002/jia2.26100

**Published:** 2023-05-23

**Authors:** Noah Mancuso, Gordon Mansergh, Rob Stephenson, Keith J. Horvath, Sabina Hirshfield, Jose A. Bauermeister, Mary Ann Chiasson, Martin J. Downing, Patrick S. Sullivan

**Affiliations:** ^1^ Department of Epidemiology, Rollins School of Public Health Emory University Atlanta Georgia USA; ^2^ Division of HIV Prevention Centers for Disease Control and Prevention Atlanta Georgia USA; ^3^ Department of Systems, Populations and Leadership, School of Nursing and The Center for Sexuality and Health Disparities University of Michigan Ann Arbor Michigan USA; ^4^ Department of Psychology San Diego State University San Diego California USA; ^5^ STAR Program, Department of Medicine SUNY Downstate Health Sciences University Brooklyn New York USA; ^6^ Department of Family and Community Health School of Nursing University of Pennsylvania Philadelphia Pennsylvania USA; ^7^ Department of Epidemiology, Mailman School of Public Health and Division of Infectious Diseases, Department of Medicine Columbia University Irving Medical Center New York New York USA; ^8^ Department of Psychology Lehman College, CUNY Bronx New York USA

**Keywords:** HIV, HIV testing, self‐testing, mobile apps, men who have sex with men, digital divide

## Abstract

**Introduction:**

The United States Centers for Disease Control and Prevention currently recommends HIV screening at least annually among sexually active gay, bisexual and other men who have sex with men (MSM), but only half report being tested in the past year in the United States. As HIV self‐test kits are becoming more available around the United States via web and app‐based interventions, it is important to understand who is willing and able to order them. This analysis sought to better understand predictors of free HIV self‐test kit utilization among MSM in M‐cubed, an HIV prevention mobile app intervention trial in Atlanta, Detroit and New York City.

**Methods:**

We conducted an exploratory secondary analysis of self‐report and in‐app data collected from the intervention arm of the M‐Cubed study from 24 January 2018 to 31 October 2019. Behavioural, demographic and other potential predictors of HIV self‐test ordering were identified from Social Cognitive Theoretical underpinnings of the app, and from the literature. Significant predictor variables in bivariate analyses were considered for inclusion in the empiric multivariable model. Demographic variables chosen a priori were then added to a final model estimating adjusted prevalence ratios (aPR).

**Results:**

Over half of the 417 intervention participants ordered an HIV self‐test kit during the study. In bivariate analyses, ordering a kit was associated with HIV testing history, plans to get tested and reported likelihood of getting tested. In the final model, participants were more likely to order a kit if they reported plans to get tested in the next 3 months (aPR = 1.58, 95% CI: 1.18–2.11) or had not tested for HIV in the past 3 months (aPR = 1.38, 95% CI: 1.13–1.70). There was no difference in HIV self‐test kit ordering by income, race/ethnicity or age.

**Conclusions:**

HIV testing is an important tool in ending the HIV epidemic and must be accessible and frequent for key populations. This study demonstrates the effectiveness of HIV self‐test kits in reaching populations with suboptimal testing rates and shows that self‐testing may supplement community‐based and clinical testing while helping overcome some of the structural barriers that limit access to annual HIV prevention services for MSM.

## 1 INTRODUCTION

New HIV diagnoses in the United States have declined overall in the past 10 years [1], but the goal of the Ending the HIV Epidemic in the US (EHE) initiative to reduce new HIV diagnoses by 90% by 2030 will likely not be met without more population‐specific prevention efforts [2]. The number of incident HIV diagnoses among individuals aged 25–34 years increased from 2009 to 2018 [3]; as of 2019, gay, bisexual and other men who have sex with men (MSM) comprised 69% of new HIV diagnoses [1, 4]. Though the number of new diagnoses decreased among non‐Hispanic White (hereafter referred to as White) MSM, numbers remained the same for non‐Hispanic Black or African American (Black) MSM and increased for Hispanic/Latino (Hispanic) MSM.

The United States Centers for Disease Control and Prevention (CDC) currently recommends HIV screening at least annually among sexually active MSM [5], yet in national surveys, only half of MSM report being tested in the past 12 months [6]. Distributing free HIV self‐test kits via online services and mobile apps has shown to be associated with increased testing [7, 8], especially among populations who may not be able to access clinic‐ or community‐based testing [9, 10, 11]. However, concerns remain about equitable access and willingness to use web and app‐based interventions for ordering HIV self‐test kits based on evidence of a “digital divide.” The “digital divide” is a gap in technology use and uptake observed among older adults, people with low socio‐economic status and minority racial and ethnic groups due to a lack of financial resources, mistrust or reluctance and low digital literacy [4, 12, 13]. As these same groups are also bearing the brunt of the HIV epidemic in the United States, it is important to question if mHealth interventions will bridge these gaps or further exacerbate disparities.

There is strong evidence that HIV self‐testing is acceptable among MSM in the United States with benefits, including convenience, privacy, ease of use and the ability to reach undertested populations [14, 15]. The National HIV Behavioral Surveillance survey found that among MSM who tested for HIV, 31% reported testing in a non‐clinical setting—including a mobile unit, HIV counselling site or self‐test [16]. MSM in the United States are more than 50% more likely to test for HIV when given the option of self‐testing, which leads to the identification of more persons newly diagnosed with HIV and can increase cost‐savings [17]. Ongoing studies are evaluating the effectiveness of mailing kits to Black and Hispanic MSM, such as the Implementation of Rapid HIV Self‐Testing Among MSM Project (iSTAMP) [18, 19] and the TRUST study [20]. A CDC‐supported national HIV self‐test kit distribution programme, marketed towards MSM through messages and embedded links in gay dating applications, reached equitable populations of Hispanic MSM, but not Black MSM [11]. Given that some MSM may benefit from HIV screening more often than annually based on behaviours that may increase their chances of getting HIV, it is important that self‐directed options for more frequent screening be available, especially to younger, Black and Hispanic MSM. However, little is known about the extent and variation of the use of app‐based interventions to access HIV self‐test kits in the population of MSM.

The Mobile Messaging for Men (M‐cubed) randomized control trial [21, 22] showed that providing MSM a mobile app that included the opportunity to order HIV self‐test kits was associated with a doubling of the rate of HIV testing. To understand the extent to which MSM utilize free HIV self‐test kits via app‐based interventions and to determine if various characteristics of MSM are associated with increased kit ordering, we used intervention data from M‐cubed with the aim of identifying predictors of ordering kits during a 3‐month intervention period.

## 2 METHODS

### 2.1 Study design

Data collected between 24 January 2018 and 31 October 2019 from the intervention arm of the M‐Cubed randomized controlled trial were used in this study [21, 22]. The M‐cubed mobile app was developed using Social Cognitive Theory (SCT), which posits that individual health behaviours are influenced by an individual's experiences, actions of others and environmental factors [23], to address multiple HIV and sexually transmitted infection (STI) prevention and care needs accounting both for risk factors (condomless anal sex without taking pre‐exposure prophylaxis [PrEP] as prescribed in the past 3 months) and for risk reduction (taking PrEP as prescribed, using condoms, or avoiding anal sex). Sexual health messaging for MSM in the intervention arm was built into app written content and videos, which included screening for HIV, and STI risk; scheduling and reminders for routine testing; PrEP and non‐occupational post‐exposure prophylaxis eligibility screeners; and commodity ordering for HIV and STI self‐test kits, condoms and lubricant. Individual informed consent was collected in person at the study enrolment visit. Eligible and consented MSM were randomized 1:1 either to the intervention group with immediate access to the M‐Cubed app for 3 months, or to the wait‐listed control group who received delayed access to the app until after final outcome assessments. This research was reviewed and approved by the Emory University institutional review board (protocol IRB00087684). The methods, full baseline questionnaire, and primary results from the M‐cubed app randomized control trial have previously been described [21, 22].

### 2.2 Measures

The outcome of our analysis was ordering an HIV self‐test kit during the 3‐month intervention period, which we collected from in‐app data and dichotomized as ever ordered or never ordered. Baseline data were collected by self‐report on demographics, variables related to SCT, and potentially associated variables from the literature were collected and considered as predictors.

**Figure 1:**
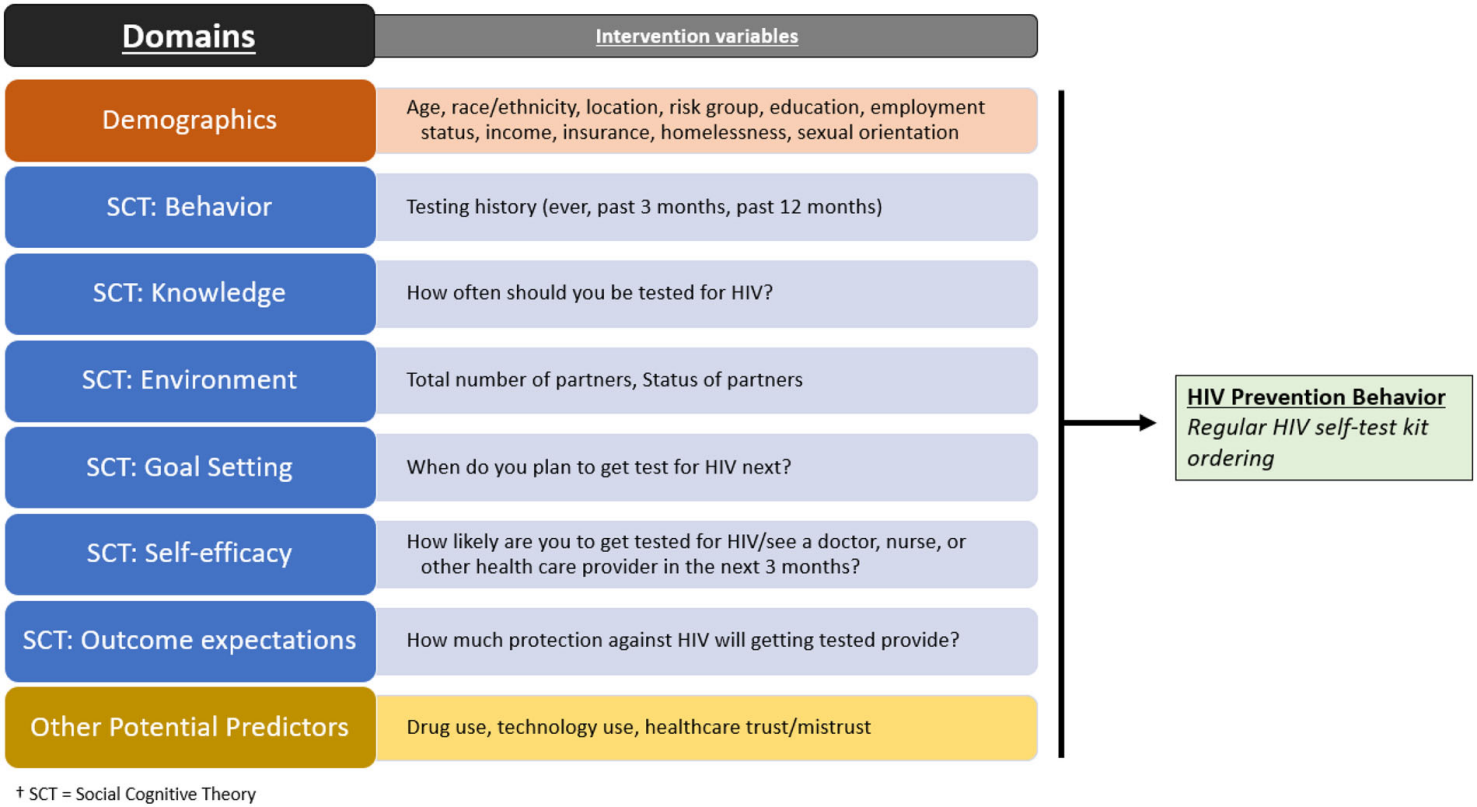
Relationship between the baseline M‐Cubed survey variables used in the analysis, mapped to their SCT domains, and the HIV prevention behaviour outcome of ordering an HIV self‐test kit.

Because the M‐cubed app was conceptualized using SCT and these variables were hypothesized to be predictive of HIV self‐test kit ordering, an initial evaluation of the subset of variables used in our analysis was mapped to SCT domains (Figure 1) [23, 24]. These measures included six domains from the SCT framework (behaviour, knowledge, environment, goal setting, self‐efficacy and outcome expectations). Behaviour was assessed by asking participants if they had tested for HIV ever, in the past 12 months, and in the past 3 months. Knowledge was assessed by asking participants how often one should be tested for HIV. For the Environment variable, both main partners (defined as someone a participant had lived with or had seen a lot and to whom they felt a special emotional commitment for at least 3 months) and casual partners (defined as someone the participant had sex with but did not feel committed to or did not know very well) were summed and then categorized into three levels to describe the number of partners (0, 1–2 and 3 or more). Goal Setting was based on when participants planned to get tested for HIV in the near future. The Self‐Efficacy variable was derived from participants’ self‐reported likelihood to actually get tested for HIV. Outcome Expectations were assessed as how much protection participants think testing provides against acquiring HIV.

The potentially associated variables from the literature with HIV testing or HIV self‐test kit ordering that were considered in the bivariate analysis included the Drug Use Disorders Identification Test (DUDIT), Modified Technology Use Scale and Health Care Mistrust Scale [25, 26, 27, 28]. All measures and their respective levels used for the purpose of this study are presented in Table 1 as well as in Figure 1 with their respective domains.

**Table 1:** Factors impacting HIV kit ordering among gay, bisexual and other men who have sex with men in Atlanta, Detroit and New York, 2018

	Total (*n* = 417)	Ordered (*n* = 219)	Did not order (*n* = 198)	Bivariate analysis
Variable	*N* (col %)	*N* (row %)	*N* (row %)	Unadjusted PR (95% CI^a^)
**DEMOGRAPHICS** **Age**				
18–25 years	112 (27)	59 (53)	53 (47)	Reference
26–30 years	109 (26)	68 (62)	41 (38)	1.18 (0.94, 1.49)
31–40 years	105 (25)	51 (49)	54 (51)	0.92 (0.71, 1.20)
>40 years	91 (22)	41 (45)	50 (55)	0.86 (0.64, 1.14)
**Race/Ethnicity**				
White, non‐Hispanic	213 (51)	117 (55)	96 (45)	Reference
Black/African American, non‐Hispanic	90 (22)	46 (51)	44 (49)	0.93 (0.74, 1.18)
Hispanic/Latino	61 (15)	30 (49)	31 (51)	0.90 (0.67, 1.19)
Other^b^	53 (12)	26 (49)	27 (51)	0.89 (0.66, 1.21)
**Site**				
Atlanta	142 (34)	75 (53)	67 (47)	Reference
New York	137 (33)	60 (44)	77 (56)	0.83 (0.65, 1.06)
Detroit	138 (33)	84 (61)	54 (39)	1.15 (0.94, 1.41)
**Risk group**				
High risk^c^	215 (52)	119 (55)	96 (45)	Reference
Low risk^d^	202 (48)	100 (50)	102 (50)	0.89 (0.74, 1.07)
**Education**				
High school graduate or GED and below	43 (10)	23 (53)	20 (47)	Reference
Some college, Associate or Technical Degree	111 (27)	60 (54)	51 (46)	1.01 (0.73, 1.40)
Bachelor's Degree/College Degree	144 (35)	73 (51)	71 (49)	0.95 (0.69, 1.31)
Any post‐graduate studies	119 (29)	63 (53)	56 (47)	0.99 (0.71, 1.37)
**Work**				
Employed full time	258 (62)	142 (55)	116 (45)	Reference
Employed part‐time	90 (22)	48 (53)	42 (47)	0.97 (0.78, 1.21)
Unemployed, unable to work and other	69 (17)	29 (42)	40 (58)	0.76 (0.57, 1.03)
**Income**				
$0–$14,999	80 (19)	40 (50)	40 (50)	Reference
$15,000–$29,999	81 (19)	47 (58)	34 (42)	1.16 (0.87, 1.55)
$30,000–$49,999	87 (21)	47 (54)	40 (46)	1.08 (0.81, 1.45)
$50,000–$74,999	86 (21)	41 (48)	45 (52)	0.95 (0.70, 1.30)
$75,000 or more	82 (20)	43 (52)	39 (48)	1.05 (0.78, 1.42)
Missing	1 (0)	1 (100)	0 (0)	
**Insurance**				
Yes	355 (85)	184 (52)	171 (48)	Reference
No	61 (15)	35 (57)	26 (43)	1.11 (0.87, 1.41)
Missing	1 (0)	0 (0)	1 (100)	
**Homeless** **^e^**				
No	391 (94)	207 (53)	184 (47)	Reference
Yes	25 (6)	12 (48)	13 (52)	0.91 (0.60, 1.38)
Missing	1 (0)	0 (0)	1 (100)	
**Sexual orientation**				
Gay or homosexual	356 (85)	193 (54)	163 (46)	Reference
Bisexual, heterosexual or other	61 (15)	26 (43)	35 (57)	0.79 (0.58, 1.07)
**SOCIAL COGNITIVE THEORY (DOMAINS)** **Ever tested for HIV (Behaviour)**				
Yes	374 (90)	192 (51)	182 (49)	Reference
No	43 (10)	27 (63)	16 (37)	1.22 (0.95, 1.57)
**HIV test in the past 12 months (Behaviour)**				
Yes	315 (76)	154 (49)	161 (51)	Reference
No	102 (24)	65 (64)	37 (36)	1.30 (1.08, 1.57)
**HIV test in the past 3 months (Behaviour)**				
Yes	200 (48)	87 (44)	113 (57)	Reference
No	217 (52)	132 (61)	85 (39)	1.40 (1.16, 1.69)
**How often should you be tested for HIV? (Knowledge)**				
Once a year or less	63 (15)	34 (54)	29 (46)	Reference
Every 6 months	136 (33)	74 (54)	62 (46)	1.01 (0.77, 1.33)
Every 3 months or more frequently	203 (49)	104 (51)	99 (49)	0.95 (0.73, 1.24)
Other^f^	15 (4)	7 (47)	8 (53)	0.86 (0.48, 1.56)
**Total number of partners in the past 3 months (Environment)**				
3 or more	216 (52)	102 (47)	114 (53)	Reference
1–2	177 (42)	106 (60)	71 (40)	1.27 (1.05, 1.53)
0	24 (6)	11 (46)	13 (54)	0.97 (0.61, 1.53)
**HIV status of partner(s) (Environment)**				
All believed negative	266 (64)	137 (52)	129 (48)	Reference
At least one positive	24 (6)	13 (54)	11 (46)	1.05 (0.71, 1.55)
At least one unknown, none known positive	102 (24)	57 (56)	45 (44)	1.09 (0.88, 1.34)
No partners reported	25 (6)	12 (48)	13 (52)	0.93 (0.61, 1.42)
**When do you plan to get tested for HIV next? (Goal Setting)**				
Within next 3 months	278 (67)	159 (57)	119 (43)	1.60 (1.18, 2.17)
Within next 4–12 months	84 (20)	30 (36)	54 (64)	Reference
More than a year, not planning, don't know	55 (13)	30 (55)	25 (45)	1.53 (1.05, 2.22)
**How likely are you to get tested for HIV in the next 3 months? (Self‐Efficacy)**				
Definitely or Probably NOT likely/Does not apply to me	43 (10)	15 (35)	28 (65)	Reference
Somewhat, Probably, or Definitely Likely	373 (89)	203 (54)	170 (46)	1.56 (1.03, 2.37)
Missing	1 (0)	1 (100)	0 (0)	
**How likely are you to see a doctor, nurse or other healthcare provider in the next 3 months? (Self‐efficacy)**				
Somewhat, Probably, or Definitely Likely	371 (89)	192 (52)	179 (48)	Reference
Definitely or Probably NOT likely/Does not apply to me	44 (11)	26 (59)	18 (41)	1.14 (0.88, 1.49)
Missing	2 (0)	1 (50)	1 (50)	
**How much protection does getting tested for HIV provide in preventing HIV? (Outcome Expectations)**				
0–49	85 (20)	46 (54)	39 (46)	Reference
50–69	108 (26)	63 (58)	45 (42)	1.08 (0.84, 1.39)
70–94	89 (21)	42 (47)	47 (53)	0.87 (0.65, 1.17)
95–100	103 (25)	56 (54)	47 (46)	1.00 (0.77, 1.31)
Missing	32 (8)	12 (38)	20 (63)	
**OTHER POTENTIAL PREDICTORS** **Drug use in the past 3 months (DUDIT)**				
No	273 (65)	144 (53)	129 (47)	Reference
Yes	144 (35)	75 (52)	69 (48)	0.99 (0.81, 1.20)
**Use of phone for dating or hookup apps? (Technology Use)**				
Yes	273 (65)	149 (55)	124 (45)	Reference
No	144 (35)	70 (49)	74 (51)	0.89 (0.73, 1.09)
**How many hours do you spend on the cell phone per day? (Technology Use)**				
<2 hours	75 (18)	38 (51)	37 (49)	Reference
2–3 hours	101 (24)	54 (53)	47 (47)	1.06 (0.79, 1.41)
3–4 hours	94 (23)	50 (53)	44 (47)	1.05 (0.78, 1.41)
> 4 hours	144 (35)	75 (52)	69 (48)	1.03 (0.78, 1.35)
Missing	3 (1)	2 (67)	1 (33)	
**Healthcare Trust/Mistrust Scale**				
>30	93 (22)	44 (47)	49 (53)	Reference
26–30	69 (17)	37 (54)	32 (46)	1.13 (0.83, 1.54)
16–25	173 (41)	94 (54)	79 (46)	1.15 (0.89, 1.48)
0–15	73 (18)	39 (53)	34 (47)	1.13 (0.83, 1.53)
Missing	9 (2)	5 (56)	4 (44)	

^a^ Wald CI with a test statistic of *z* = 1.96.

^b^ Includes Middle Eastern, Brazilian, Indian and Asian Pacific Islander.

^c^ Condomless anal sex and not taking PrEP as prescribed in the past 3 months.

^d^ No condomless anal sex in the past 3 months, or condomless anal sex while taking PrEP as prescribed in the past 3 months.

^e^ Living on the street, in a shelter, a Single Room Occupancy hotel (SRO), temporarily staying with friends or relatives, or living in a car at any time in the past 12 months.

^f^ “Other” option selected, but fill in the blank was left empty.

### 2.3 Statistical analysis

Only HIV‐negative participants assigned to the intervention (*n* = 417) were included in this analysis. Predictor variables that yielded a *p*‐value ≤ 0.05 in bivariate analyses were considered for inclusion in the empiric model. We used forward, backward and stepwise selection methods to derive the reduced empiric model. Variables with significant collinearity were assessed in separate models and when similar explanatory values were found, the final model was chosen based on the variable most likely to be influenced by the intervention. We included two age categories for MSM within the 18 and 30 age range due to the heterogeneity of risk [29] and to create groups with comparable numbers for a total of four age categories (i.e. 18–25, 26–30, 31–40, >40 years old). Because age and race are defining components of disparities in the HIV epidemic, we decided a priori to retain them in the final model. Income and number of sexual partners were also retained in the final model based on differences in baseline testing within groups and evidence from the literature as indicators of healthcare access and environmental risk, respectively [30, 31, 32, 33, 34]. Other variables from the app and SCT domains were not forced into the final model. Log‐binomial models were used to estimate prevalence ratios (PRs). We conducted all analyses in SAS (version 9.4; SAS Institute Inc., Cary, NC).

## 3 RESULTS

Among HIV‐negative participants in M‐cubed who were randomly assigned to the intervention (*n* = 417), 22% identified as Black and 15% as Hispanic (Table 1). The mean age was 33 years (standard deviation [SD] = 12) with the majority (64%) having a bachelor's degree or higher. Baseline self‐reported testing rates in the past 12 months were high (76%) and differed significantly by age group (66% for ages 18–25 years; 72% for ages 26–30 years; 83% for ages 31–40 years; 81% for ages over 40 [*p* < 0.05]), income level (64% for less than $14,999; 68% for $15,000–$29,999; 80% for $30,000–$49,999; 80% for $50,000–$74,999; 85% for $75,000 or more [*p* < 0.01]) and number of partners (66% for no partners, 71% for 1–2 partners; 84% for 3 or more partners [*p* < 0.001]). There were no significant differences in baseline testing by race and ethnicity (74% for White, non‐Hispanic; 76% for Black, non‐Hispanic; 78% for Hispanic; 75% for Other [*p* = 0.90]). About one in five participants had high levels of medical mistrust at baseline, which differed significantly by race and ethnicity (6% for White, non‐Hispanic; 52% for Black, non‐Hispanic; 36% for Hispanic; 25% for Other [*p* < 0.0001]), income (30% for less than $14,999; 24% for $15,000–$29,999; 24% for $30,000–$49,999; 24% for $50,000–$74,999; 12% for $75,000 or more [*p* < 0.05]) and number of partners (25% for no partners, 22% for 1–2 partners; 16% for 3 or more partners [*p* < 0.05]). Baseline technology use, a composite score of reported app use, was high among participants, but only differed significantly by race and ethnicity (69% for White, non‐Hispanic; 53% for Black, non‐Hispanic; 64% for Hispanic; 74% for Other [*p* < 0.05]). A little over half of the participants (53%) ordered an HIV self‐test kit during the 3‐month intervention period. Of those who ordered a kit, 5% ordered more than one kit during the intervention period. Data were only available for ordering of kits, not on testing behaviour of participants after kits were ordered.

In bivariate analyses, demographic variables, such as age or race and ethnicity, were not associated with HIV self‐test kit ordering. Among SCT variables, Behaviour (self‐reported HIV testing history), Goal Setting (plans to test for HIV) and Self‐Efficacy (self‐report likelihood of testing) were all associated with ordering a kit. Behaviour (self‐reported HIV testing history) was associated with the outcome in the past 12 months (unadjusted PR = 1.30, 95% CI: 1.08–1.57) and in the past 3 months (unadjusted PR = 1.40, 95% CI: 1.16–1.69). To reduce collinearity, we retained HIV testing only in the past 3 months in the final model (Table 2). For Goal Setting, compared to those who reported at baseline that they plan to test in the next 4–12 months, participants who reported that they planned to test in the next 3 months were 60% more likely to order an HIV self‐test kit during the intervention period. Similarly, for Self‐Efficacy, participants who were somewhat, probably or definitely likely to get tested in the next 3 months were 56% more likely to order a kit than those who said they were definitely or probably not likely to get tested or who said that testing did not apply to them. Due to significant collinearity between Goal Setting (plans to test for HIV) and Self‐Efficacy (self‐report likelihood of testing), we only included Goal Setting in the final model due to the larger explanatory value of the variable and it being more programmatically related to the M‐cubed intervention.

**Table 2:** Adjusted prevalence ratios for final model factors predicting HIV kit ordering among gay, bisexual and other men who have sex with men in Atlanta, Detroit and New York, 2018

	Empiric model	Final model
Variable	Adjusted PR (95% CI)	Adjusted PR (95% CI^a^)
**Age**		
18–25 years		Reference
26–30 years		1.17 (0.93–1.47)
31–40 years		0.99 (0.74, 1.30)
>40 years		0.89 (0.64, 1.24)
**Race/Ethnicity**		
White, non‐Hispanic		Reference
Black/African American, non‐Hispanic		0.92 (0.73, 1.16)
Hispanic/Latino		0.89 (0.67, 1.18)
Other^b^		0.83 (0.62, 1.11)
**Income**		
$0–$14,999		Reference
$15,000–$29,999		0.97 (0.73, 1.29)
$30,000–$49,999		1.11 (0.82, 1.50)
$50,000–$74,999		0.87 (0.64, 1.19)
$75,000 or more		1.12 (0.80, 1.57)
**HIV test in the past 3 months**		
Yes	Reference	Reference
No	1.42 (1.17, 1.71)	1.38 (1.12, 1.70)
**When do you plan to get tested for HIV next?**		
Within next 3 months	1.58 (1.17, 2.14)	1.58 (1.18, 2.11)
Within next 4–12 months	Reference	Reference
More than a year, not planning, don't know	1.31 (0.90, 1.91)	1.21 (0.83, 1.75)
**Total number of partners in the past 3 months**		
3 or more		Reference
1–2		1.15 (0.95, 1.41)
0		0.93 (0.60, 1.46)

^a^ Wald CI with a test statistic of *z* = 1.96.

^b^ Includes Middle Eastern, Brazilian, Indian and Asian Pacific Islander.

Other SCT variables related to the design of the M‐cubed app (e.g. Knowledge, Outcome Expectations and Environment) were not associated with HIV self‐test kit ordering in the study population. Additionally, novel variables related to HIV self‐testing through harm reduction for participants engaging in online sexual partners seeking behaviours—such as the use of phone for dating apps and hours spent on the phone per day—were not significant predictors in the bivariate analysis in the study population, despite associations observed elsewhere [35, 36, 37].

In our final multivariable model in Table 2, the SCT variables of Behaviour (self‐reported HIV testing history) and Goal Setting (plans to test for HIV) remained significant predictors of HIV self‐test kit ordering. Participants who had not been tested for HIV in the past 3 months were 38% more likely to order a kit during the study intervention than those who had been tested in the past 3 months. Participants who had plans to get tested at baseline in the next 3 months were 58% more likely to order a kit than those who planned to get tested at baseline in the next 4–12 months. No other variables in our final model were significantly associated with HIV self‐test kit ordering.

## 4 DISCUSSION

Using data from the M‐cubed randomized control trial, we assessed factors associated with ordering an HIV self‐test kit and found that accessing free HIV self‐test kits via a mobile phone app can reduce baseline disparities in testing by age, income and number of sexual partners while overcoming barriers of technology use and medical mistrust by different racial and ethnic groups. This is important, as testing is one of the four strategies of the EHE initiative, with a focus on expanding testing to reach populations most vulnerable to HIV acquisition [38]. It is also the first step in the “status‐neutral” continuum to engage people in HIV care regardless of status [39]. Additionally, we found that self‐reported HIV testing history and plans to test for HIV were the strongest predictors of self‐testing among all variables considered, indicating that the M‐Cubed app may help better align MSM testing intentions with behaviour and reach populations who reported not recently testing for HIV.

Participants in this study had high rates of HIV testing in concordance with CDC recommendations [5]. About three in four MSM reported being tested in the past 12 months, which is higher than national surveys but similar to findings from other comparable urban settings [6, 40]. Our bivariate analysis found that individuals who reported at baseline not testing for HIV in the past 3 months or in the past 12 months were more likely to order HIV self‐test kits. This variable remained statistically significant in our final model. Controlling for all other variables, participants who had not tested in the past 3 months were more than 30% more likely to order kits than those who had tested in the past 3 months. This reaffirms earlier findings [7, 8, 9, 10, 11] that self‐testing is effective in reaching undertested populations. By offering an opportunity to reach MSM not already routinely testing according to CDC guidelines, HIV self‐test kits may supplement, although not completely replace testing in clinical settings.

Our study found that plans to test for HIV in the next 3 months were associated with ordering kits both in bivariate analyses and in our final model. Those who planned to get tested for HIV in the next 3 months at baseline were more than 1.5 times more likely to order a kit than those who planned to get tested for HIV in the next 4–12 months. These findings are consistent with previous literature on the significant association between intentions and regular HIV testing [41, 42]. This highlights the importance of programmes that target improving HIV testing plans, like the *Tu Amigo Pepe* campaign in Seattle [43], and especially programmes with linguistic and cultural relevance for MSM populations with suboptimal testing rates who are over‐represented in the HIV epidemic. It is important to note that intentions do not always align with behaviour. This is particularly true among marginalized groups who may experience structural barriers or mistrust, due to the historical mistreatment of these groups in medical practice, which may impede acting on intentions [44, 45, 46]. By making self‐test kits freely available online, thus lowering barriers from cost, mistrust and stigma, we may be able to better align intentions with behaviour by increasing testing among populations with suboptimal testing rates.

Distributing HIV self‐test kits aims to remove barriers to physical access, which may help mitigate structural factors that are known to drive inequities in the use of HIV prevention and sexual health services [47, 48, 49]. Thus, we are equally interested in associations that might exist based on levels of access to HIV prevention services in other studies but were not observed in this analysis. For example, research points to higher rates of late HIV diagnosis in neighbourhoods with higher income inequality and socio‐economic deprivation when compared to the rates of late HIV diagnosis in neighbourhoods with low inequality [50]. Rates of PrEP uptake among Black MSM were lower or comparable to rates of PrEP uptake among White MSM, even though Black MSM are over‐represented in the HIV epidemic [51, 52]. Baseline testing rates in our analysis are concurrent with other survey findings, indicating that the proportion of MSM testing for HIV in the past year was equal in Black and White MSM, despite higher HIV prevalence among Black MSM [53]. In our analysis, baseline testing rates did differ by age, number of sexual partners and income. However, when kits were offered for ordering without charge through the intervention, we did not find differences in ordering by age, number of partners, income or race. This suggests that offering free self‐test kits may provide HIV testing opportunities that can overcome some of the structural barriers that are believed to limit access to prevention services among MSM. In a large national survey of MSM, self‐reported HIV testing in the past year was lower among younger MSM. Our results suggest that ordering of HIV self‐test kits might be a useful approach to close the gap in annual testing for younger MSM compared to the CDC testing recommendations for MSM [5, 54, 55]. Although there were no significant relationships between income, race, ethnicity, or age and ordering kits, these findings may be significant in practice, in that they provide evidence for offering free HIV self‐test kits to equitably bridge the digital divide in HIV testing access.

This study had several limitations. Although the study oversampled from minority race and ethnic populations, the sample was skewed towards MSM with higher education and high baseline testing rates. The study also only sampled from three large, urban US cities thus missing out on important potential differences in behaviours in urban and rural settings. These selection biases impair the external generalizability of the study, as risk factors and HIV self‐test ordering behaviours may differ by populations. Second, baseline survey data used in this study relied on self‐report, so misclassification of the predictors may have occurred due to social desirability bias for certain variables like testing history and number of sexual partners. Third, ordering a kit does not necessarily mean that a participant used the test or used it properly. Test kit use has been shown to vary in similar studies anywhere from 52% to 90% [7, 57]. As seen in other HIV self‐testing studies, a small percentage of participants may also order kits for friends or partners [7, 17, 28], thus our findings may overestimate actual testing behaviour and uptake among participants themselves. Fourth, the intervention period was only over 3 months. As testing is currently recommended for MSM once annually, the intervention may not have captured the true rate of ordering kits among MSM that occur during an entire year. Fifth, the kits were offered for free in the context of this study. Although the CDC is currently piloting programmes to distribute free HIV self‐test kits, our findings may not be replicable outside of the research setting. Sixth, the study did not test any varying intensity of messaging among participants, which means data were not available to assess how messaging intensity impacts a participant's ordering behaviours. Lastly, the study was conducted prior to COVID‐19. Since the pandemic brought an increase in clinical care being provided remotely and increased access to self‐testing for COVID‐19, it is possible that the acceptability and usage of HIV self‐tests may have changed significantly.

The CDC recently updated its recommendations for HIV screening in MSM [5], and it is clear from samples of urban MSM [56] and national samples [53] of MSM that better mechanisms to promote frequent HIV testing are needed. The distribution of HIV self‐test kits to MSM is associated with a large increase in annual testing frequency, and men who receive a positive screening test with a mailout kit have the same rate of linkage to confirmatory testing and HIV care as men tested in conventional testing venues [53]. The CDC has recently reported on a direct‐to‐consumer model of distributing HIV self‐test kits, finding that the effort reached substantial numbers of MSM who had never tested for HIV, or who had not tested in the past year [11]. As mobile apps become more prevalent and available for HIV prevention and sexual health, self‐testing may become a normative option for testing within app features. The findings from this analysis provide evidence for the equitable scale‐up of mHealth interventions to overcome significant differences in baseline testing and to reach undertested populations. The M‐Cubed app used for this analysis was recently designated as a risk reduction best evidence‐based intervention by the CDC, which allows state health departments and HIV prevention grantees to receive support from the CDC to implement the intervention [57, 58]. While the hope is to bring the app to scale in the United States, it should be noted that many challenges exist in the translation of mHealth research into practice. To date, no apps funded by the National Institutes of Health (NIH) are currently accessible to members of the general public and most HIV prevention apps on the market were not developed by academic or public health entities [59]. Significant resources and effort must be invested to ensure the successful translation of app‐based health technology to practice in the United States and more broadly, as the distribution of HIV self‐test kits is a novel but important additional implementation strategy [60] to help MSM achieve the CDC recommendation of HIV testing at least annually.

## 5 CONCLUSIONS

HIV testing is the cornerstone of all the tools to end the HIV epidemic and it is the starting point of the status‐neutral continuum for people at risk for HIV and people living with HIV [39]. Ending the HIV epidemic will not happen unless HIV testing for key populations is made accessible and frequent, which requires innovative solutions like M‐Cubed. According to our data, offering HIV self‐test kits to MSM at substantial risk for acquiring HIV should be a critical component of a system of testing opportunities that support MSM to test at least annually, and more often when indicated. This self‐directed intervention may supplement community‐based and clinical testing, while helping overcome barriers to frequent HIV testing and helping MSM with their HIV testing goals.

## COMPETING INTERESTS

Authors Mancuso, Mansergh, Stephenson, Horvath, Hirshfield, Bauermeister, Chiasson, Downing and Sullivan report no competing interests.

## AUTHORS’ CONTRIBUTIONS

GM, RS, KJH, SH, JAB, MAC, MJD and PSS designed the research. PSS, GM, RS and SH oversaw the study implementation. NM, GM and PSS analysed the data. NM and PSS wrote the initial draft of the manuscript. NM, GM, RS, KJH, SH, JAB, MAC, MJD and PSS provided critical input to the manuscript draft. The final manuscript was approved by NM, GM, RS, KJH, SH, JAB, MAC, MJD and PSS.

## FUNDING

This work was supported by a Cooperative agreement from CDC (CDC U01PS004977). This work was supported by the Center for AIDS Research at Emory University (NIH P30AI050409).

## DISCLAIMER

The findings and conclusions are solely the responsibility of the authors and do not necessarily represent the official position of the U.S. Centers for Disease Control and Prevention.

## Data Availability

The data that support the findings of this study are available on request from the corresponding author.
